# Philosophical Transactions of the Royal Society of London. Vol. LXXII. For the Year 1782. Part II

**Published:** 1783

**Authors:** 


### THE
### LONDON MEDICAL JOURNAL,
### For JULY, AUGUST, and SEPTEMBER, 1783.

### SECTION I.

### BOOKS.

I. *Philosophical Transactions of the Royal Society of London.* *Vol.* LXXII. *for the year* 1782. *Part* II. 4*to.* *London,* 1783, 158 pages with 9 copper-plates.

1. *AN attempt to make a thermometer for measuring the higher degrees of heat, from a red heat up to the strongest that vessels made of clay can support. By* Josiah Wedgewood; *communicated by Sir* Joseph Banks, *Bart.* P. R. S. ——A measure for the higher degrees of heat, such as the common thermometers afford for the lower ones, has long been a desideratum both with the philosopher and the practical ar-

 tist.

tist. The ingenious author of the paper before us after having attentively confidered this subject, difcovered in argillaceous bodies a property which feemed to anfwer the purpofe he was in fearch of. This property is the *diminution of their bulk by fire.*

He has found that this diminution begins to take place in a low red heat; and that it proceeds regularly, as the heat increafes, till the clay becomes vitrified, and confequently to the utmoft degree that crucibles, or other veffels made of this material, can fupport.

If, therefore, we can procure at all times a clay fufficiently apyrous or unvitrefcible, and always of the fame quality in regard to contraction by heat; and if we can find means of meafuring this contraction with care and minute accuracy, he thinks we fhall be furnifhed with a meafure of fire fufficient for every purpofe of experiment or bufinefs.

Of all the forts he has hitherto tried, fome of the pureft Cornifh porcelain clays feem the beft adapted, both for fupporting the intenfity, and meafuring the degrees of fire.

For preparing and applying this material to thermometric purpofes, the clay is firft to be wafhed over, and whilft in a dilute ftate, paffed through through a fine lawn. In the lawns uſed by our author the interſtices are each leſs than the 100,000 part of an inch. The clay is then to be dried and put up in boxes, to ſecure it from the effects of air and moiſture.

The dry clay is to be ſoftened, for uſe, with about two-fifths of its weight of water; and formed into ſmall pieces, in little moulds of metal, ſix-tenths of an inch in breadth, with the ſides pretty exactly parallel, (this being the dimenſion intended to be meaſured) about five-tenths of an inch deep, and one inch long. To make the clay deliver eaſily, it will be neceſſary to oil the mould, and make it warm.

Theſe pieces, when perfectly dry, are put into another iron mould or gage, conſiſting only of a bottom, with two ſides, five-tenths of an inch deep; to the dimenſions of which ſides the breadth of the pieces is to be pared down.

For meaſuring the diminution which they are to ſuffer from the action of fire, another gage is made of two pieces of braſs, twenty-four inches long, with the ſides exactly ſtraight, divided into inches and tenths, fixed five-tenths of an inch aſunder at one end, and three-tenths at the other, upon a braſs plate; ſo that one end of the thermometric pieces, when pared down in the iron gage, will juſt fit to the wider end. If this piece has diminiſhed in the fire one-fifth of its bulk, it will then paſs on to half the length of the gage; if diminiſhed two-fifths, it will go on to the narroweſt end; and in any intermediate degree of contraction, if the piece be ſlid along till it reſts againſt the converging ſides, the degree at which it ſtops will be the meaſure of its contraction, and conſequently of the degree of heat it has undergone.

The ſcale of this new thermometer commences at a red heat, fully viſible in day-light; and the greateſt heat that Mr. Wedgewood has hitherto obtained in his experiments is 160°. The greateſt degree of heat that could be produced in a common ſmith's forge was 125°. Swediſh copper melts at 27°, ſilver at 28°, and gold at 32°. Braſs is in fuſion at 21°. The welding heat of iron is from 90° to 95°. Caſt-iron was found to melt at 130°. The thermometer pieces begin to acquire a porcelain texture at 110°, but it is remarkable that notwithſtanding this, their contraction, on further augmentations of the heat, proceeds regularly as before, up to the higheſt degree of fire Mr. Wedgewood has been able to produce.

To

To obviate any objection which might be made to these thermometers on a supposition that the clay employed for this purpose may not be always easily procurable in sufficient quantity, and on moderate terms, Mr. Wedgewood observes, that in Cornwall there are beds of this clay, inexhaustible, and in too many hands to be monopolized. If this should not prove satisfactory, he offers to the Royal Society, and adds, that he shall think himself honoured by their acceptance of a sufficient space in a bed of this clay to supply the world with thermometer pieces for numerous ages.

In the course of the paper the ingenious author offers several other observations and cautions relative to the making and using of this thermometer. What we have said will be sufficient to convey to our readers an idea of the nature and utility of the invention.

In an appendix is given an analysis of the clay of which the thermometer pieces are formed.

II. *Account of the organ of hearing in fish. By* John Hunter, *esq.* F.R.S.—In this paper the ingenious author does not profess to give a full account of the organ of hearing in any one fish, or of the varieties in different fish, but only of the the organ in general. Its ſituation, he obſerves, is at the ſides of the cranium, but the cranium itſelf makes no part of the organ, as it does in the quadruped and bird. In ſome fiſh this organ is wholly ſurrounded by the parts compoſing this cavity, which in many is cartilaginous, the ſkeleton of theſe fiſh being like thoſe of the ray kind; but even in others, as in cod, ſalmon, &c. whoſe ſkeleton is bone, this part is ſtill cartilaginous.

It appears to grow in ſize with the animal, and is much more ſimple in fiſh than in quadrupeds, birds and amphibious animals.—It varies in different orders of fiſh; but in all it conſiſts of three curved tubes, all of which unite with one another. This union in ſome, as in the cod, &c. forms only a canal, and in others as in the ray kind a pretty large cavity. The whole is compoſed of a kind of cartilaginous ſubſtance, very hard or firm in ſome parts, and which in ſome fiſh is cruſted over with a thin bony lamella, ſo as not to allow them to collapſe.—Each tube deſcribes more than a ſemicircle. This reſembles in ſome reſpect what we find in moſt other animals, but differs in the parts being diſtinct from the ſcull. Mr. Hunter obſerves, however, that the turtle and the crocodile have a ſtructure ſomewhat ſimilar to this, and that the intention is the ſame, as their ſculls make no part of the organ.

Two of the ſemicircular canals are ſimilar to one another, may be called a pair, and are placed perpendicularly; the third is not ſo long; in ſome it is placed horizontally, uniting as it were the other two at their ends or terminations. In the ſkait it is ſomething different, being only united to one of the perpendiculars.

The two perpendiculars, by one arm of each uniting, form one canal, while the other two arms or horns have no connection with each other, and the arms of the horizontal unite with the other two arms of the perpendicular near the entrance into the common canal or cavity.—— Near the junction of theſe canals with the common cavity, they are ſwelled out into round bags, becoming there much larger.

In the cod they terminate, as has been obſerved, in one canal, which in theſe fiſh is placed upon the additional cavity or cavities in which there is one or more bones. In the jack which has two cavities, we find in one of thoſe cavities two bones, and in the other only one. In the ray there is only a chalky ſubſtance, ſimilar lar to what is found in the ears of amphibious animals.

At this union of the two perpendiculars in ſome fiſh enters what may be called the external meatus. This is the caſe, we are told, with all the ray kind, but it is not every genus or ſpecies of fiſh that has the external opening.

The nerves of the ear paſs outwards from the brain, and appear to terminate at once on the external ſurface of the ſwelling of the ſemicircular tubes above deſcribed. They do not appear to paſs through thoſe tubes ſo far as to get on the inſide, as is ſuppoſed to be the caſe in quadrupeds. Mr. Hunter therefore ſuſpects that the lining of thoſe tubes in the quadruped is not nerve but a kind of internal perioſteum.

The paper concludes with an account of an experiment made by the author in 1762, when in Portugal, and which he thinks ſhews that fiſh are much affected by ſound. This experiment conſiſted in firing a gun behind ſome ſhrubs near a fiſh-pond, ſo that there might not be the leaſt reflection of light. The inſtant the report was made, the fiſh, who had before been ſeen ſwimming about, darted to the bottom of the pond, raiſing at it were a cloud of mud, but in about five minutes they began to appear again.

III. *Some farther considerations on the influence of the vegetable kingdom on the animal creation. By* John Ingenhousz, *counsellor to the court and body physician to the emperor,* F. R. S. &c.

Dr. Ingenhousz here vindicates his doctrines from the attack * of Dr. Priestley, who was led by his experiments to assert that plants exposed to the sunshine do not generate dephlogisticated air, as Dr. Ingenhousz had supposed, but merely purify the air already contained in the water and in the atmosphere.

Several experiments made in a hothouse of the botanic garden at Vienna in the winter of 1782 are related, but for these we must refer our readers to the paper itself. Dr. Ingenhousz flatters himself that these experiments prove the justness of his assertion, that vegetables really throw out air in the sunshine; but if there should still be philosophers who do not think the facts he has related sufficiently decisive, he advises them to be present, at least once, at the most beautiful scene which they will behold, when a leaf of an *Agave Americana*, cut in two or three pieces, is immersed in a glass bell or jar full of pump water, inverted and exposed

* See the 2d volume of this Journal, p. 5.

to the ſun in a very fair day, in the middle of the ſummer, when this plant is in its full vigour; and when they ſhall have ſeen thoſe beautiful and continual ſtreams of air which ruſh from ſeveral parts of this vegetable, principally from the white internal ſubſtance of it, he will be anſwerable for their laying aſide all further doubt about the truth of his doctrine. In a fair day, he tells us, he has often got from a ſingle leaf of this plant above 150 cubic inches of dephlogiſticated air of the fineſt quality.

Dr. Ingenhouſz having demonſtrated, as he thinks, that vegetables diffuſe through our atmoſphere, in the ſunſhine, a continual ſhower of this truly vital air, and that plants, immerſed in water, impregnate it fully with this ſame ſalubrious air, adviſes us to derive ſome benefit from this new diſcovery, by making uſe of veſſels of water, in which ſome leaves of vegetables have been expoſed in the ſunſhire; by placing ſuch veſſels in our rooms; by ſtirring the water, and by ſprinkling our floors with it, inſtead of uſing common water for this purpoſe; and by placing within our houſes, inſtead of flower-pots, diſhes containing ſome *conferva rivularis*, a plant eminent for yielding dephlogiſticated air, and which is to be met with almoſt every where,

ſhooting

ſhooting forth with the utmoſt luxuriancy in all water baſons, in all tubs and veſſels in which water is kept.

The other papers contained in this volume are *an account of two mineral ſubſtances, viz. the Rowley rag ſtone and the toad ſtone*; by William Withering, M. D.—*New fundamental experiments upon the colliſion of bodies*; *by Mr.* John Smeaton, F. R. S.—*Proceedings relative to the accident by lightning at Heckingham.*——*Account of a new electrometer*; *by Mr.* Abraham Brook.—*A new method of inveſtigating the ſums of infinite ſeries*; *by the Rev.* S. Vince, A.M.—*A new method of finding the equal roots of an equation, by diviſion*; *by the Rev.* John Hellins, *curate of Conſtantine, in Cornwall*—*A microſcopic deſcription of the eyes of the Monoculus Polyphemus* Linnæi; *by Mr.* William André, *ſurgeon.*

